# Systematic Review of Prognostic Factors for Return to Work in Workers with Sub Acute and Chronic Low Back Pain

**DOI:** 10.1007/s10926-016-9666-x

**Published:** 2016-09-19

**Authors:** Ivan A. Steenstra, Claire Munhall, Emma Irvin, Nelson Oranye, Steven Passmore, Dwayne Van Eerd, Quenby Mahood, Sheilah Hogg-Johnson

**Affiliations:** 1 Institute for Work and Health, Toronto, ON Canada; 2 Ted Rogers School of Management, Ryerson University, 350 Victoria Street, Toronto, ON M5B 2K3 Canada; 3 University of Manitoba, Winnipeg, MB Canada

**Keywords:** Disability, Sick leave, Disability evaluation, Review, Systematic, Prognosis

## Abstract

*Purpose* We systematically reviewed the evidence on factors that predict duration of sick leave in workers after 6 weeks low back pain (LBP) related sick leave. We hypothesized that different factors affect the duration of the leave depending on the time away from work. *Methods* The review occurred in seven phases: (1) developing the central question, (2) conducting the literature search, (3) identifying relevant publications, (4) quality appraisal, (5) data extraction, (6) evidence synthesis, and (7) knowledge translation. We searched for studies that reported episodes of LBP and sick leave that lasted more than 6 weeks. All included studies reported at least one prognostic factor where return to work was the outcome. *Results* We identified twenty-two relevant publications. The impact of pain, functional status and radiating pain seems to change with duration of work disability. Workers’ recovery expectations remain important after 6 weeks. Modified duties are rarely studied in later phases of work disability. Depression/mental health did not appear to be an important factor in later phases. Workplace physical factors remain important. There is insufficient evidence that pain catastrophising and fear avoidance are predictive factors in later phases. There was moderate evidence for age in the later phases. Functional capacity and claim related factors were supported by some evidence. *Discusion* Physical demands in the workplace are preventing workers from getting back to work in a timely fashion across phases. The psychosocial work environment is understudied in later phases. Overall, we cannot conclude that prognostic factors change over time.

**Electronic supplementary material:**

The online version of this article (doi:10.1007/s10926-016-9666-x) contains supplementary material, which is available to authorized users.

## Background

Delayed return to work (RTW) is associated with high compensation and treatment costs. In the United States (US) indirect costs of low back pain (LBP) are estimated to be more than US $50 billion per year [1], in the United Kingdom (UK) US $11 billion [2] and in the Netherlands almost US $5 billion [3]. Hoy et al. [4] state that LBP causes more global disability than any other condition and that there is an urgent need for further research to better understand LBP across settings.

Frank et al. [5] proposed a model that classifies three stages in the work disability process: Acute (0–6 weeks); Subacute (6–12 weeks); and Chronic (12+ weeks). Over the course of the work disability process some workers return to work, while others remain off work. Time is needed to recover from injury, but over time RTW could become complicated by a number of factors. Factors that might be predictive at an early stage of RTW might differ from those that are important at a later stage.

We previously published systematic reviews on prognostic factors for duration on sick leave due to acute LBP (with duration of 0–6 weeks) [6, 7]. These reviews showed that there was strong evidence that the following factors had an association with the duration of sick leave: recovery expectations, radiating pain (injury severity), pain (self report), disability (self report), workplace physical factors, and provider type. There was also strong evidence that lifestyle and pain catastrophising had no association with duration of sick leave. Moderate evidence was found for modified duties, workplace psychosocial factors, claim-related factors and content of treatment and there was moderate evidence for no association of previous injury and clinical examination. There was insufficient evidence for age, education, language barriers, depression/mental health, fear avoidance beliefs, work relatedness, workplace-organizational factors and the process of treatment.

This study extends the scope of the previous reviews by systematically reviewing the evidence on factors that predict duration of time away from work at the sub acute and the chronic stage of a LBP related episode of time away from work. The first hypothesis was that there are factors related to LBP, to the worker, to the job and to the psychosocial environment that influence duration of an episode of sick leave.

The second hypothesis was that in the sub acute and even more so in the chronic phase, psychosocial issues will likely become more prominent compared to the acute phase.

## Methodology

### Classifying Prognostic Factors

LBP is considered to be a multidimensional problem. A framework proposed by Loisel et al. [8] further elaborates the structure of the International Classification of Functioning, Disability and Health (ICF) [9–11] to also include factors related to the workplace, healthcare and workers’ compensation environment. By applying this framework we further distinguished between predictive factors related to the LBP, the worker, the job and workplace, and the psychosocial environment, specifically to health care services and the workers compensation insurer. These theoretical frameworks structured our analyses and aided in clear reporting to stakeholder groups.

### Classifying Outcomes

The concept of time away from work is highly dependent on legislation and locally-used jargon. In North America, “time away from work”, “time on disability benefits” or “disability” is used to define time away from work. In Europe, the phrases “sick leave” and “RTW” are used more often, since disability is used to define functional limitations (for instance as measured by the Roland Morris Disability Questionnaire). These differences were recognized in our search strategy [6].

### Search Strategy

We used an updated search strategy from previous reviews [12–14] (see Online Appendix I) in Medline (OVID), EMBASE (OVID) and PsycINFO (OVID) from inception of each database to 2012. The search was constructed in three broad categories: (1) Prognosis terms, (2) Back Pain terms, and (3) Work/Return To Work terms. The terms within each category were combined with an OR Boolean operator and then the three categories were combined with an AND Boolean operator. The search therefore captured references with at least one term in each of the three categories. As each database is unique, the search was customized accordingly to best utilize the controlled vocabularies of each. Search yields were combined and duplicates were removed. We reviewed the search yield for studies on LBP [12], prognosis [15] and work and stratified the results for each phase of work disability. The references list of all relevant articles and recently published systematic reviews were screened for additional publications. An in-depth comparison of search strategies [15, 16] has shown that our search strategy was broad enough to capture as much relevant literature as possible.

### Selection of Studies

Two reviewers independently selected studies that met similar inclusion and exclusion criteria as in our previous reviews [6, 17], except for the disability phase:

1. Studies that included subjects with an episode of LBP and sick leave, with duration of more than 6 weeks at inclusion of cohort;
2. Studied the relation between at least one prognostic factor and outcome; and
3. Measured outcomes in absolute terms (rate), relative terms (odds ratio, rate ratio, hazard ratio), survival curve or duration of sick leave.

First, titles and abstracts were screened, followed by possibly relevant full articles. A third reviewer resolved disagreements if consensus between two reviewers could not be reached.

The third reviewer (IS or SHJ) used his/her knowledge and experience in the field of prognosis research to weigh the different view of assessing studies for suitability. The initial reviewers reached consensus in most cases and the third reviewer only had to be consulted in a minimal number of cases where reviewers erred on the side of caution.

### Quality Assessment

Two reviewers independently scored the quality of included studies. The quality was appraised using a tool developed in our previous reviews [7] (see Online Appendix II). Item 24 asked the reviewer for a general appraisal of study quality using a 11 point VAS scale. This item was not used in the assessment of study quality, because of a lack of agreement with the overall scale score. The third reviewer (IS or SHJ) used his/her knowledge and experience in the field of prognosis research to weigh the different view of assessing studies for suitability. The initial reviewers reached consensus in most cases and the third reviewer only had to be consulted in a minimal number of cases where reviewers erred on the side of caution.

### Data Extraction

Due to heterogeneity in studies we did not conduct a meta-analysis. The evidence for each prognostic factor was therefore presented in a descriptive manner.

The information extracted from each study included definition of prognostic factor and outcome, country, setting, association estimate, sample size. Risk of RTW was recalculated to the risk of no RTW. This means that a ratio larger than 1 means a delay in time until RTW.

### Levels of Evidence

Relevant studies were grouped by prognostic factors and the level of evidence for each prognostic factor was determined by into consideration the quality ratings of each study and the consistency of findings across studies in terms of significance and direction of association across the different studies examining each particular prognostic factor. The criteria for describing the level of evidence for each prognostic factor is based on van Hoogendoorn et al. [18] rating system and is consistent with our previous reviews on prognosis in RTW in the acute phase of LBP [6, 19]:

- *Strong evidence* consistent findings in multiple high quality studies.
- *Moderate evidence* consistent findings in one high quality study and one or more lower quality studies, or in multiple lower quality studies.
- *Insufficient evidence* only one study available or inconsistent findings in multiple studies.

## Results

The initial search yielded 5027 research papers, after duplicates were removed. After the screening of all titles and abstracts, 939 papers were retrieved for a more full text review. Seventy-eight publications met all of the inclusion criteria. Sixteen publications were from the chronic phase, six were from the sub acute phase, 37 were from the acute phase and 19 studies were either in populations from different phases or did not report the duration of sick leave.

**Fig. 1:**
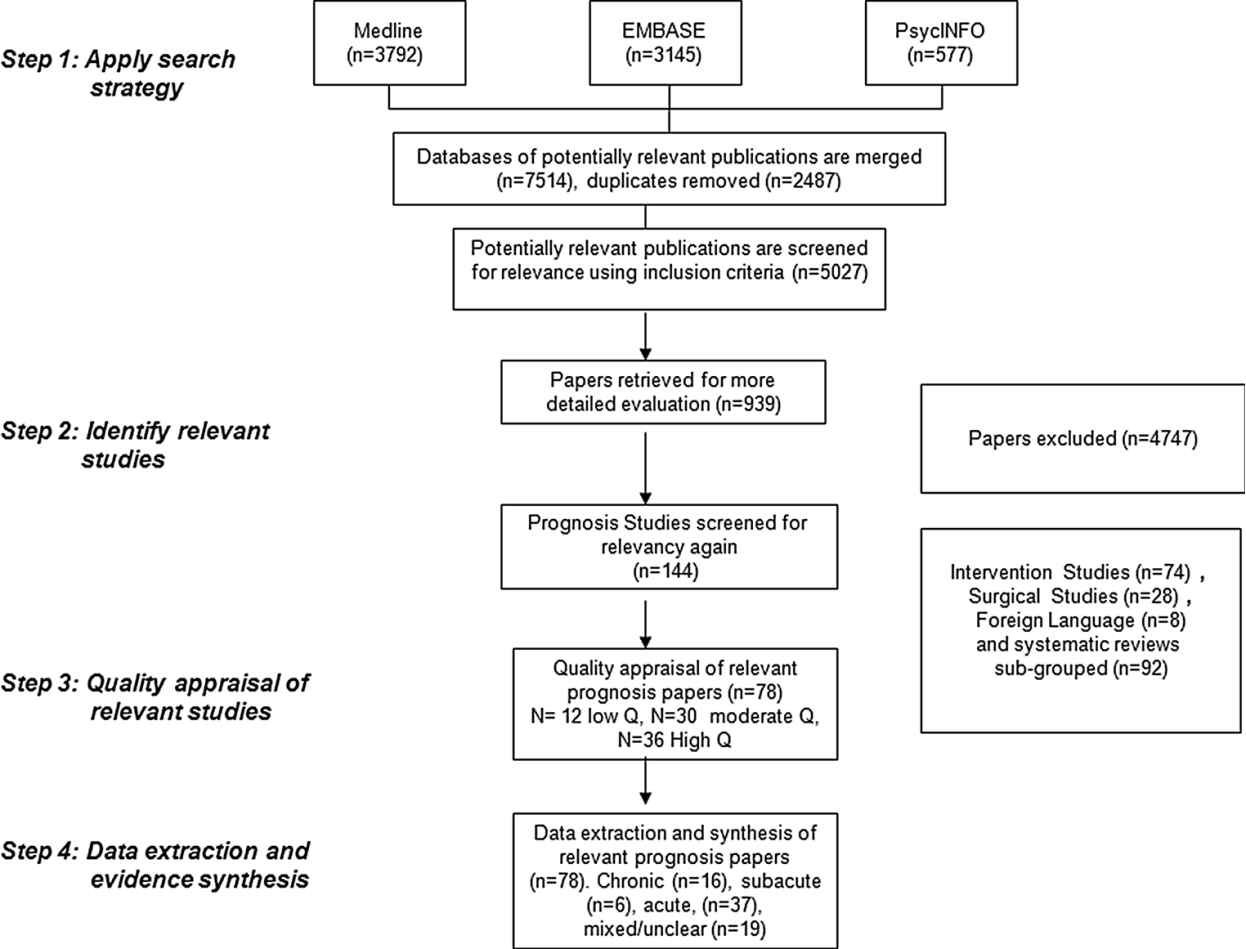
Flowchart chronicling the search process

Publications that included cases from the sub acute phase were from Canada (4), the USA (1) and Norway (1). Publications that included cases from the chronic phase were from Canada (3), the USA (6), Norway (1), Netherlands (3) and from an international study in Denmark, Germany, Israel, Sweden, the Netherlands, and the United States (3). (See Fig. 1.)

In the sub acute phase the mean quality score was: 15.5 (range 14–19). In the chronic phase the mean quality score was: 14.8 (range 9–21). Five of these studies were high quality (QA score > 16), 13 were of moderate quality (QA score between 13 and 16), and seven were of lower quality (QA < 13).

481 prognostic factors were considered were studied across all phases. Prognostic factors were grouped in a number of team meetings into clinical, personal psychosocial, work related psychosocial, and claim related prognostic factors. Within each category, factors or tools measuring the same or very similar constructs (for instance different methods to report on physical demands) were merged resulting in 43 different constructs that we report on. See Table 1 for detailed characteristics of the included studies.

**Table 1:** Characteristics of included studies

First author	Year	Country	Setting	Outcome definition	Final sample size	N factors	Quality score
Sub acute phase: 6–12 weeks since first day of injury/sick leave							
Reme (22)	2009	Norway	Secondary care	RTW	175	12	19
Truchon (23)	2010	Canada	WC	RTW	296	9	17
Gross (70)	2004	Canada	WC	RTW	114 & 132 (2 cohorts)	3	15
Joy (24)	2001	USA	Secondary care	RTW	115	6	14
Truchon (25)	2005	Canada	WC	RTW	321	4	14
Schultz (34)	2004	Canada	WC	RTW	214	9	14
Chronic phase >12 weeks since first day of injury/sick leave							
Gross (36)	2005	Canada	Tertiary care	Benefits	228 & 210 (2 cohorts)	8	21
Gauthier (19)	2006	Canada	Tertiary care;	Benefits	255	7	19
Okurowski (26)	2003	USA	WC	Benefits	962	4	19
Gross (71)	2005	Canada	OHS	RTW	45	4	18
Storheim (33)	2005	Norway	Secondary care	Benefits	93	3	18
Anema (72)	2004	Multinational	OHS; WC	RTW	1631	3	17
Blackwell (20)	2004	USA	WC	Benefits	227	9	17
Van der Giezen (27)	2000	ISSA study: Netherlands	WC	RTW	298	12	16
Koopman (73)	2004	Netherlands	Tertiary care	RTW	51	5	15
Anema (29)	2009	ISSA study	WC	RTW	2444	19	14
Mayer (74)	2001	USA	Tertiary care	RTW	1052		13
Hansson (28)	2000	ISSA study	Occupational health services	RTW	2106	3	11
Vendrig (21)	1999	Netherlands	Secondary care	RTW	137	16	11
Hazard (30)	1991	USA	Secondary & tertiary care	RTW	195 (1 year), 69 (2 years)	6	11
Gallagher (75)	1989	USA	Primary care; WC	RTW	140	6+ interactions	9
Gatchel (31)	1994	USA	Tertiary care	RTW	152	1	8

*WC* Worker’s compensation, *OHS* Occupational health service, *RTW* Return to work, *Benefits* workers compensation/disability benefits

### Evidence on Prognostic Factors

We present the results on the evidence for association for factors in the chronic phase followed by those from the subacute phase according to the categories we determined.

A summary of the evidence on prognostic factors is presented in Table 2.

**Table 2:** Prognostic factor evidence table

Prognostic factor	Phase	Evidence	Level of evidence
*Clinical prognostic factors*			
Sex	Chronic	2H, 1 M, 1L	Moderate for negative association with male sex
	Subacute	2H, 1 M, 1 M	Strong for no association
Age	Chronic	2H, 1H, 2 M, 2L, 1L	Moderate for negative association
	Subacute	1H, 1 M	Moderate for negative association
Comorbidity	Chronic	1 M	Insufficient
Diagnosis	Chronic	1L	Insufficient
Radiating	Subacute	1H	Moderate for no association
Pain Intensity	Chronic	1H, 2 M, 2L	Moderate for negative association
	Subacute	2H, 1 M	Strong for no association
Functional status	Chronic	2 M, 1L	Moderate for negative association
	Subacute	2H, 2 M	Strong for no association
Functional status-FCE	Chronic	1H, 1 M, 2L	Moderate for positive association
	Subacute	2 M (2 cohorts, one publication)	Moderate for positive association
Pain observation	Subacute	1 M	Insufficient
Delay in referral	Chronic	4H, 1L	Strong for negative association
Intervention	Chronic	1 M, 1H	Moderate for positive association
	Subacute	1H, 2 M	Moderate for positive association
Health	Chronic	2 M, 1L	Moderate for positive association
	Subacute	1 M	Insufficient
Lifestyle	Chronic	1L	Insufficient
*Psychosocial prognostic factors*			
Expectation of RTW	Chronic	2 M, 1L	Insufficient
	Subacute	1H, 1 M	Moderate for positive association
Fear avoidance/Pain catastrophising/Cognitive appraisal/Coping	Chronic	1H, 1 M,1L	Moderate evidence for negative association*
	Subacute	1H, 1 M	Moderate evidence for negative association*
Distress	Subacute	1H	Insufficient
Depression	Chronic	1H, 1L	Moderate for no association
Mental Health	Chronic	2L, 2L	Insufficient
*Social workplace prognostic factors*			
SES	Chronic	2H, 1 M,1L	Strong evidence for positive association
	Subacute	1H	Insufficient
Physical demands	Chronic	1H, 1 M	Moderate evidence for positive association
	Subacute	1H	Insufficient
Modified duties	Chronic	1 M	Insufficient
Social support	Chronic	1H	Insufficient
	Subacute	1 M	Insufficient
Job satisfaction	Chronic	1 M	Insufficient
Attorney involvement	Chronic	2H	Strong evidence for negative association
Worker’s compensation	Chronic	2H, 1 M, 1L	Strong evidence for negative association

*H* High quality, *M* Moderate quality, *L* Low quality, *SES* Socio economic status, *Heterogeneity in measures

### Results on Clinical Prognostic Factors

#### Sex and Age

*Chronic phase* There is moderate evidence of negative association between male sex and RTW from 1 medium quality [20] and one high quality study [21]. Notably, one high quality study [22] and one low quality study [23] did not find an association between sex and RTW. However, since they did not find an association between female sex and RTW, this is not a contradictory finding and could be the result of small sample sizes.

*Sub acute phase* There is strong evidence for no association between sex and RTW in the sub acute phase based on two high quality studies [24, 25] and a medium quality study [26]. Only one medium quality study [27] found a negative association with male sex.

*Chronic phase* There is moderate evidence for a negative association between older age and RTW from a high quality study [24] and a medium quality study [26].

Most studies reported a negative association between older age and RTW, although not all were statistically significant [21–23].

*Subacute phase* There is moderate evidence for older age from one high quality study [28], two medium quality publications [20, 29] and one lower quality publication [30] that reported a negative association between age and RTW.

#### Pain and Function

*Chronic phase* Radiating pain was not studied in the selected studies. There is moderate evidence that pain intensity has a negative association with RTW in the chronic phase from one high quality study [21], one moderate quality study [29–31] and two lower quality studies [23, 32]. One lower quality study did not report on pain [33]. There is moderate evidence for a negative association between function and RTW from one lower quality [23] and two medium quality studies [20, 34]. With respect to the results of functional capacity evaluation, there is moderate evidence for a positive association with RTW, based on one high quality study (cardiovascular fitness) [35] and one medium quality study (trunk flexibility) [20], however two lower quality studies [23, 32] found no significant association with RTW.

*Sub acute phase* There is moderate evidence for no association of radiating pain with RTW [25]. There is strong evidence for no association with function from two high quality studies [24, 25] and two medium quality studies [36, 37]. There is strong evidence for no association between pain and RTW as well from two high quality studies [24, 25] and one medium quality study [36]. There is moderate evidence for a positive association of a higher score on a functional capacity evaluation (FCE) and RTW from one publication reporting on two cohorts of injured workers [37]. There was one study that observed a negative association between pain observation and RTW [25].

#### Treatment Related Clinical Factors

*Chronic phase* There is strong evidence from 4 high quality studies [21, 22, 28, 38] and one lower quality study [23] that a delay in referral to intervention was associated with a delay in RTW. One high quality study [22] found a positive association of insurer mandated rehabilitation and one medium quality study [31] found a positive association of several medical interventions (surgery between 4 and 12 months, pain medication and exercise therapy), which results in moderate evidence for a positive association between “intervention” and RTW.

*Sub acute phase* There is moderate evidence for a positive association between intervention and RTW from one high quality (prior physiotherapy) [24] and two medium quality studies (time in work hardening program and stretching) [26, 27].

#### Health Related Clinical Factors

*Chronic phase* There is moderate evidence for health measures on RTW from two medium quality (general health and physical function) [29, 35] and one lower quality (social function in Sweden and USA) [30] studies.

*Subacute phase* There was insufficient evidence for a positive association of health on RTW because only one study [36] found a positive association between this factor and RTW in this phase.

*Chronic phase* A medium quality study [32] found no association between lifestyle (smoking) and RTW.

### Results on Personal Psychosocial Factors

#### Recovery Expectations

*Chronic phase* Researchers from one international study [29–31] reported inconsistently on the one item question from the work ability index [39] that asks about expectations of RTW. We conclude that there is insufficient evidence for recovery expectations in the chronic phase.

*Sub acute phase* One high quality study [24] found a positive association of expectations of RTW with RTW, even though only the 3 months mark was statistically significant. Another medium quality study [36] also found a negative association of low expectations with RTW. We consider this as moderate evidence for the association between recovery expectations and RTW.

#### Pain Catastrophising, Fear Avoidance, Coping

*Chronic phase* One medium quality study [35] found a negative association between FAB-Q and time on benefits. The high quality study from Gauthier et al. [21] found no association of fear of movement on time on benefits. One high quality study [21] reported a negative association between pain catastrophising and time on benefits. Another medium quality study [20] found a negative association between coping and RTW. We argue there is moderate evidence for a negative association for the concept of fear of movement, since different, but conceptually similar, measures were used in a limited number of studies.

*Sub acute phase* Again, different, but conceptually similar, measures were used in a limited number of studies, resulting in limited evidence for all these factors. One high quality study [25] found a rather strong association of the score on the Fear Avoidance Beliefs Questionnaire (FAB-Q) and RTW (odds ratios of 5 and 3). This study [25] also found a negative association of cognitive appraisal on RTW. One medium quality publication from the same first author [27] found a negative association between pain catastrophising and RTW. Again, there is moderate evidence for a negative effect of the fear of movement concept.

#### Distress, Depression, Mental Health

*Sub acute phase* Only distress was examined in this phase in one high quality study [25], which reported a negative association with RTW. There is insufficient evidence for the association between distress and RTW in the sub acute phase because only one study considered this category of factors.

*Chronic phase* One high quality [21] and one lower quality [23] studies found no statistically significant association between depressive symptoms and RTW, resulting in moderate evidence for no association for this factor. The high quality study examined 7 constructs in a population with 98 events, which indicates sufficient power for this study [40].

*Chronic phase* Three low quality studies [23, 32, 33] examined the association between mental health and RTW. One of the studies [23] reported a negative association, while the other two found no effect of mental health on RTW. Therefore, there is insufficient evidence for a negative association between mental health and the outcome.

### Results on Work Related Psycho-Social Factors

#### Socioeconomic Status, Physical Demands, and Modified Duties

These factors are reported in the same section because they are related. Workers that are classified as having lower socio economic status (SES), often have more physically demanding jobs. Modified duties are often used to (temporarily) mitigate the negative associations of physically demanding work. Unfortunately, none of the studies in the review measured these factors simultaneously.

*Sub acute phase* One high quality study [24] found a positive association between lower physical demands and RTW. The same study found no association between education and RTW. In summary there is insufficient evidence for an association with RTW in the sub acute phase, due to a limited number of high quality studies.

*Chronic phase* One high quality [22], one lower quality [30] and one medium quality [31] publications (all from the ISSA study) showed a positive association between lower physical demands and faster RTW. Due to the limited number of high quality studies, there is moderate evidence for physical demands on RTW in the chronic phase. We found strong evidence for SES, although it was measured in rather different ways in different studies. One high quality study [28] reported a negative association between language barriers and RTW. Another high quality publication [22] found a positive association of higher education with RTW. A medium quality publication [29] found a positive association between being a breadwinner and RTW. One lower quality publication [23] only reported a non significant association for education. One medium quality publication [31] reported positive associations between modified duties and RTW. However, due to the limited number of studies there is insufficient evidence for the association between modified duties and RTW in the chronic phase.

#### Social Support, Skill Discretion, Job Satisfaction

*Sub acute phase* one medium quality publication [36] reported no association between social (co-worker) support and RTW. That same study reported a negative association between skill discretion and RTW.

*Chronic phase* One high quality study [22] reported a non significant (positive) association of being married on RTW, but also reported a not statistically significant association between skill level and RTW. A medium quality publication [29] reported a positive association between job satisfaction and RTW, oddly the other publications from this study [30, 31] did not report on it.

In summary, *t*here is insufficient evidence due to the lack of high quality studies for all of the work related psychosocial factors in both the sub acute and chronic phases.

### Claim Related Factors

*Chronic phase* Two high quality studies reported a negative association between attorney involvement and RTW [22, 28], which results in strong evidence for the association between attorney involvement and RTW. Anema et al. [31] in their cross jurisdictional (medium quality) study, found that workers compensation policies and practice are associated with RTW outcomes. However, since only one study looked into policies and practices, this factor is supported by insufficient evidence.

*Sub acute phase* One medium quality study [36] in the sub acute phase found a negative association between Workers Compensation Board and employer response and RTW. This results in insufficient evidence to support this factor due to a lack of high quality studies.

## Discussion

Our first hypothesis was that there are factors related to LBP, to the worker, to the job and to the psychosocial environment that influence duration of an episode of sick leave. The results presented in Table 2 show that factors within the clinical, psychosocial and workplace categories are associated with RTW. Understanding these factors can help practitioners dealing with patients during the RTW process. There does not seem to be consensus between researchers on a core set of prognostic factors that should be included in prognostic studies in LBP and work disability in particular. While some may argue for the use of meta-analysis, like we did in our first review [6], we deemed meta-analysis inappropriate for this review because of the lack of consensus on adjustment of confounders. Moreover, studies measured factors in different ways, there was inconsistency in reporting methods, and a large variability in quality of the studies.

Our second hypothesis was that in the sub acute and even more so in the chronic phase, psychological and social issues would likely become more prominent compared to the acute phase. This hypothesis cannot be confirmed, mainly due to the lack of high quality studies and a lack of consensus among researchers on what to measure, how to measure, and how to analyze the associations. In our previous reviews [6, 17] we found strong evidence for no association of ‘pain catastrophising scale’ with RTW. We hypothesized that pain catastrophising might play a role at a later stage in the work disability process. However, there are not enough high quality studies to go beyond moderate evidence for any of the pain catastrophising and fear avoidance factors in later phases.

### Limitations of the Literature

The psychosocial work environment is clearly understudied in later phases. There has been lack of consensus among researchers on how to measure psychosocial constructs and how to analyze the data based on the available theoretical models [41]. Considering the theoretical underpinnings of the fear avoidance model (FAB) [41], straightforward predictive analysis might not be appropriate and techniques that take the complexity of concepts and their interrelationships in the FAB model into account might be preferred.

In the acute phase, we found strong evidence for an association between radiating pain—distinctly different from ‘non-specific’ low-back pain—and RTW [17]. Surprisingly, this factor was only examined in one study [25] from the sub acute phase and in none from the chronic phase. More research seems warranted based on the importance of this factor in the acute phase.

Unlike in our previous review [17], workplace factors were often not considered in the later phases. The related factors: SES, physical demands and modified duties were examined in a number of studies and, despite the crudeness of measures the results show some association with RTW.

For treatment related factors and for the effect found for modified duties, it should be noted that a prognostic study is not the most appropriate study design to examine effectiveness of interventions. Results on effectiveness of interventions can be biased in many ways when an appropriate control group is missing. The association of a *delay of referral* could very well be caused by immortal time bias [42] or time dependent bias [43, 44] since none of the studies applied time dependent analysis to examine this bias. Those that receive intervention are likely to differ from those who do not receive intervention either at baseline or over time.

### Strengths and Limitations of this Review

The strength of this systematic review is that we restricted the analysis to studies with a defined inception point. In an inception cohort, patients are included in the study at the same point in the course of their disease. In many studies on RTW the study population consists of a mixture of workers on sick leave and workers still at work at the time of inception. The number of patients at work during follow-up depends on both this mixture and on the presence of prognostic factors. Making inferences about the prognosis of RTW from such mixed studies may be misleading. It might be, however, that some researchers do not agree with the phases of disability [45] we used, as a framework for analysis in this review. The cut-offs of 6 and 12 weeks from the Frank et al. publication [45] are somewhat data driven: based on the median and 75^th^ percentile. Populations in different settings and jurisdictions have shown to have different medians and 75^th^ percentiles [46, 47] which could have important consequences for the effectiveness of interventions [46]. This classification of time on work related benefits has been extrapolated to outcomes of return to work and functional disability which might not always be appropriate [48].

The seminal paper by Frank et al. [45] seems to assume the outcome “end of benefits” to have a clear inception and a firm endpoint, more recent studies on recurrences [49] and trajectories [50] of low back pain have argued successfully that this is not always the case.”

For this review we used the quality assessment that we used in our previous reviews on the acute phase [6, 17] to have a consistency in methods. Based on our experience, we recognise that further research is needed in the development of a tool to assess the quality of prognostic research [51].

A prognostic study is not the most appropriate study design to examine the effectiveness of interventions. Especially because immortal time bias [42] or time dependent bias [43, 44] are not considered by the selected studies, and because those that receive intervention are likely to differ from those who do not receive intervention either at baseline or over time. Our findings on interventions should be interpreted with that limitation in mind.

Due to the time passed because of the magnitude of the review and the complicated analysis an update of the literature would be worthwhile, however we had to postpone publication because of knowledge transfer workshops and the development of a handbook for our funder. A quick screen of an updated search revealed few new high quality studies that could impact our findings in a substantial way. Some new findings on the importance opioid use in workers compensation settings are in our previous study [19], it should be noted that most of these studies were in the acute phase.

### Comparison of Factors in Different Phases

Workers’ recovery expectations seem important in later phases of work disability, despite a lack of high quality studies. It makes sense to ask an injured worker about their expectation for RTW. Unfortunately, there is no consensus among researchers on how to do so, nor have any of the questions used in the studies undergone psychometric testing. However, predictive validity was confirmed in all studies.

The impact of pain, functional status and radiating pain changes with duration when compared to the results from our review on the acute phase [17]. This is somewhat puzzling, although it could be that after some time, when the worst pain has subsided, other factors become more prominent. Workplace physical factors remain important over the entire course of work disability. Therefore, an injured worker should always be asked about the work he/she did when he/she hurt his/her back and/or what kind of job he/she will return to.

The factors ‘self report of disability’ [47, 52–60] and ‘pain intensity’ [36, 52–65] were supported by strong evidence in the acute phase, but the evidence is less clear in the sub acute phase [24, 25, 36, 37]. In the chronic phase, there is moderate evidence for a negative association of functional status [20, 23, 34] and of pain Intensity [21, 23, 29–33]. This might indicate a somewhat puzzling U-shape relationship between these factors and RTW over time. It could also be explained by the fact that studies adjust for different confounders.

One factor that was supported by strong evidence in the acute phase is the treatment-related factor: content of care [47, 52, 53]. In other words, it matters with which health-care provider the worker is in contact. We found moderate evidence for an association between treatment and RTW in the sub acute phase [24, 26, 27]. A delay in referral to intervention was associated with a delay in RTW [21–23, 28, 38]. Overall, experience with and content of treatment matters [22, 31] across all phases.

One prognostic factor that was not considered in the acute phase was the impact of functional capacity evaluations on RTW. In the sub acute phase, moderate evidence was found for an association with RTW [20, 23, 32, 35]. In the chronic phase we found moderate evidence for a positive association of a higher score on a functional capacity evaluation (FCE) on RTW [37]. It is not clear whether a full assessment of functional capacity is needed and whether it also predicts sustained RTW [66–68]. It should be noted that not only functional capacity evaluation systems were used in work disability assessments, but more traditional “objective measures” of functional capacity like a sub maximal bike ergometer test [35] and trunk flexibility [20] were also included.

In the acute phase, we found moderate evidence for no association of depression on RTW [36, 54, 58]. In the sub acute phase, a negative association between distress and RTW was reported [25]. In the chronic phase, one high quality [21] and one lower quality study [23] found no statistically significant association between depressive symptoms and RTW, resulting in moderate evidence for no association of depressive symptoms on RTW. These findings are consistent with the findings from the acute phase, although there are only a limited number of studies available. Some injured workers might suffer from mental health issues, but scores on different questionnaires do not seem to predict RTW.

Earlier [6, 17], we found that the offer of modified duties, or workplace accommodation improved RTW outcomes [52, 58, 69]. The evidence is not as strong in later phases, mainly because the factor does not seem to be considered by many researchers [31]. Also, when considering the evidence from the intervention literature [70], modified duties should be considered for RTW of injured workers. Timing of the intervention seems best in the acute phase [6, 17], although it might also be effective in the late phases [71].

Physical demands are often measured by occupation in the acute phase [52, 54]. Those classified as having more physical jobs are slower to return to work where self-reported physical demands were not associated with RTW [17]. In the later phases, very few studies examined the factor *physical demands* resulting in insufficient evidence in the sub acute phase [24] and moderate evidence in the chronic phase [22, 30, 31]. We did find strong evidence for SES on RTW [22, 23, 28, 29]. If SES is considered a proxy for physical demands at work, the association between physical demands and RTW seems consistent across phases and should be taken into consideration in the RTW process. Future research on RTW in the later phases of work disability should examine physical demands by using more objective measures.

Job satisfaction was supported by strong evidence in our previous review [17]. It was not examined in the sub acute phase and only one publication [29] reported on it in the chronic phase. The impact of job satisfaction might diminish after a longer time away from the job; however evidence for that hypothesis is lacking.

We found insufficient evidence for an association between age and sex and RTW in acute LBP [17]. There is moderate evidence for a negative association of older age on RTW [24, 26] in the sub acute phase. In the chronic phase, most studies also reported a negative association. Across all phases, the evidence is conflicting and calendar age might not be the most appropriate measure to capture the concept.

In the sub acute phase we found strong evidence for no association of sex on RTW [24–27]. However, one medium quality study found a longer time until RTW in men [26]. There is moderate evidence for an association between male sex and RTW [20, 21] in the chronic phase. Although two studies [22, 23] did not find an association between sex and RTW, this is not contradictory and could be due to small sample sizes. Overall, the association between sex and RTW is inconsistent across phases and might be the result of gender specific workplace based exposures [72].

### Future Research

Prognostic research in work disability prevention would benefit from consensus among research and practitioners on what factors are deemed important and how they should be measured and analysed. Claim-related factors are supported by strong evidence in the chronic phase, and in all cases, are related with delays and experiences in the claims process. This factor was not considered in earlier phases [17]. Some of the claim related factors might be time dependent: they start to play a role at later stages of work disability due to negative side effects of being in the administrative and adjudicative process that happens alongside the RTW process. Further study into claim-factors seems justified.

When presenting the findings from our review to practitioners, it was clear that there is little consensus on what “psychosocial” means in research but great consensus on the importance of the construct in practise. There seems to be a clear disconnect between research and practice that should be resolved.

Understanding of the importance of different prognostic factors at various times in the RTW process can inform stakeholders about the most appropriate actions that can be taken to improve RTW outcomes. To transfer the messages from this review we have presented the findings in a number of workshops. Based on the feedback from stakeholders we are currently developing a Handbook on Prognosis of RTW in LBP for use in practise. The handbook emphasizes the role of recovery expectations and the importance of the workplace and physical demands on the job, and provides suggestions to uncover these constructs when dealing with injured workers trying to RTW. The impact of providing such information to work disability practitioners should be studied.

## Electronic supplementary material

Below is the link to the electronic supplementary material.

Online Appendix (DOCX 26 kb)
