# Supplementary material for: Systematic Review of Prognostic Factors for Return to Work in Workers with Sub Acute and Chronic Low Back Pain
Source: J Occup Rehabil. 2016 Sep 19;27(3):369–81. doi: 10.1007/s10926-016-9666-x (PMC5591348; doi:10.1007/s10926-016-9666-x)
Supplement: Supplementary file 1 — Online Appendix (DOCX 26 kb) [file 10926_2016_9666_MOESM1_ESM.docx]

Appendix I: Prognosis Search Strategy in Medline(OVID)

# 1. cohort.ti,ab.

# 2. exp Cohort Studies/

# 3. course.ti,ab.

# 4. determinat$.ti,ab.

# 5. Follow-Up Studies/

# 6. incidence/

# 7. indicat$.ti,ab.

# 8. Mortality/

# 9. mortality.fs.

# 10. predict$.ti,ab.

# 11. prognos$.ti,ab.

# 12. Prognosis/

# 13. risk/

# 14. survival analysis/

# 15. or/1-14

# 16. Back/

# 17. back.ti,ab.

# 18. back ache?.ti,ab.

# 19. backache?.ti,ab.

# 20. back pain?.ti,ab.

# 21. Back Pain/

# 22. backpain?.ti,ab.

# 23. Low Back Pain/

# 24. lumbago.ti,ab.

# 25. lumbar pain?.ti,ab.

# 26. Sciatica/

# 27. sciatica.ti,ab.

# 28. or/16-27

# 29. Absenteeism/

# 30. exp Disability Evaluation/

# 31. employee?.ti,ab.

# 32. Employment/

# 33. exp Employment/

# 34. Occupational Diseases/

# 35. Occupations/

# 36. reemployment.ti,ab.

# 37. re-employment.ti,ab.

# 38. Rehabilitation, Vocational/

# 39. (return$ adj2 work$).ti,ab.

# 40. (sick$ adj2 absence?).ti,ab.

# 41. Sick Leave/

# 42. sick leave.ti,ab.

# 43. Work/

# 44. worker?.ti,ab.

# 45. Workers' Compensation/

# 46. absenteeism.ti,ab.

# 47. alternative work.ti,ab.

# 48. (attendance adj2 work).ti,ab.

# 49. (labo?r market adj2 reentry).ti,ab.

# 50. (labo?r market adj2 re-entry).ti,ab.

# 51. lost time.ti,ab.

# 52. "lost time".ti,ab.

# 53. lost workday?.ti,ab.

# 54. (maintenance adj2 work).ti,ab.

# 55. "maintenance at work".ti,ab.

# 56. presenteeism.ti,ab.

# 57. re-employ$.ti,ab.

# 58. reemploy$.mp.

# 59. second employer program$.ti,ab.

# 60. sick$ list$.ti,ab.

# 61. suitable employment.ti,ab.

# 62. sustainable employment.ti,ab.

# 63. (stay$ adj2 work$).ti,ab.

# 64. time los$.ti,ab.

# 65. "time on benefit".ti,ab.

# 66. work$ participation.ti,ab.

# 67. (work$ adj2 re-integrat$).ti,ab.

# 68. work$ readiness.ti,ab.

# 69. (work$ adj2 re-integrat$).ti,ab.

# 70. (work$ adj2 reintegrat$).ti,ab.

# 71. work role functioning.ti,ab.

# 72. workability.ti,ab.

# 73. or/29-72

# 74. 15 and 28 and 73

# 75. limit 74 to journal article

# Appendix II: Quality assessment form

| Quality Appraisal for:  Prognostic Factors for return to work from chronic low back pain, a systematic review | | |
| --- | --- | --- |
| Title:  Primary author:  Year of publication: | | |
| *Study population* | | |
| 1 | Should this article have been excluded already based on level 1a or level 1b exclusion criteria?  Please state reason for exclusion in comments box. |  |
| 2 | Adequate description of inclusion and exclusion criteria : (Examples of important inclusion/exclusion criteria descriptions of the following, but not limited to: age, duration of symptoms, duration of sick leave, comorbidity,)  += sufficient detail to understand why participants were included or excluded  -=not sufficient detail to understand why participants were included or excluded | *+ /−* |
| 3 | Recruitment setting described adequately:  += sufficient information to understand where participants were recruited from (examples include but are not limited to: General practice, hospital, occupational setting)  -=recruitment setting not described | *+ /−* |
| 4 | Recruitment methods are adequately described:  += the timeframe of recruitment and referral methods are adequately described  -=Both timeframe and referral methods are not adequately described | *+ /−* |
| *5* | The baseline study sample (i.e., individuals entering the study) is adequately described. Key characteristics include but are not limited or restricted to: age, gender, type of work, comorbidity, duration of sick leave)(study provides enough detail to compare between studies)  += Participants entering study are adequately described in terms of key characteristics  -= Participants entering study are not described in terms of key characteristics | *+ /−* |
| *Response* | | |
| 6 | Satisfactory response rate (number of subjects who enrol in study):  += equal to or greater than 75%  -= less than 75%  ?=unclear | *+ /−/?* |
| 7 | Sufficient information on non-responders versus responders to determine if they are systematically different: positive if information presented about patient/disease characteristics of responders/non-responders or if there is no selective response.  + = no important differences between responders and non-responders  − = differences between responders and non-responders | + /− |
| *Follow-up (extent and length)* | | |
| 8 | Follow-up period sufficient in length:  += follow up period is at least 12 months  -=follow up period less than 12 months  ?=unclear | *+ /−/?* |
| 9 | Proportion loss to follow up:  += if drop-outs/loss to follow up less than 20% on the last moment of follow-up  -= if drop-outs/loss to follow up greater than or equal to 20%  ?=unclear | *+ /−/?* |
| 10 | Information completers versus loss to follow-up/drop-outs:  += if demographic/clinical information (patient/disease characteristics such as age, sex and other potential prognostic predictors) was presented for completers and those lost to follow-up/drop-outs at the main moment of outcome measurement in sufficient detail to determine if they are systematically different or no drop-outs/loss to follow-up.  -=if demographic/clinical information not presented for completers and those lost to follow up | *+ /−* |
| *Outcome* | | |
| 11 | Definition of main outcome is described:  +=The method of outcome measurement used is clearly defined  -= The method of outcome measurement used is not clearly defined | *+ /−* |
| *Prognostic factors* | | |
| 12 | Is the method of prognostic factor measurement used adequately valid and reliable to limit misclassification bias?  +=The method of prognostic factor measurement used is adequately valid and reliable to limit misclassification bias (used standardized questionnaires or standard/objective measurements were used at baseline).  -=standardized questionnaires or standard/ objective measures weren’t used for measurement of prognostic factors | *+ /−* |
| 13 | Assessment of Patient characteristics and potential clinical prognostic factor(s): (Examples include but are not limited to: age, sex, pain, functional status, duration of complaints, back complaints, physical workload)  += If clinical prognostic factors were assessed  -=If no clinical prognostic factors were assessed | *+ /−* |
| 14 | Potential psychological prognostic factor(s): (Examples include but are not limited to: depression, somatisation, distress, fear & avoidance, coping strategies, psychosocial work-related factors (social support, job decision latitude))  += If psychological prognostic factors were assessed  -=If no psychological prognostic factors were assessed | *+ /−* |
| 15 | Standardised assessment of potential social or workplace prognostic factor(s): (Examples include but are not limited to: social support, worker’s compensation, workplace organization)  += If social or workplace prognostic factors were assessed  -=If no social or workplace prognostic factors were assessed | *+ /−* |
| 16 | Did authors address potential issues surrounding missing data?  += Methods for dealing with missing data were appropriate (used multiple imputation, complete case analysis or provided a description of cases with and without missing data).  -= methods for dealing with missing data were not appropriate | *+ /−* |
| *Data presentation* | | |
| 17 | Frequencies given of main outcome measure (return to work):  += frequency, percentage or mean, median (Inter Quartile Range) and standard deviation/CI are reported of the outcome measures  -=above measures not reported | *+ /−* |
| 18 | Frequencies of all prognostic factors:  +=positive if frequency, percentage or mean, median (Inter Quartile Range) and standard deviation/CI are reported of all prognostic factors mentioned in the article  -=above measures not reported | *+ /−* |
| 19 | Appropriate analysis techniques:  += if univariate crude estimates are provided or if hazard ratios, odds ratios, relative risks or relative risk ratios are presented.  -= correlations are reported. | *+ /−* |
| 20 | Multivariate prognostic model is presented:  += attempt is made to determine a set of prognostic factors with the highest prognostic value. Positive if a manual forward stepwise procedure was used (pin <0.05; pout ≥0.10)  -= case of an analysis based on an automated forward or stepwise procedure. | *+ /−* |
| 21 | Sufficient numbers:  +=positive if the number of events in the multivariate linear regression, logistic regression and survival analysis was at least ten times the number of independent variables in the analysis. Positive if the numbers as determined a priori by a sample size calculation were achieved.  -=if the number of events is less than 10 times the number of independent variables for a multivariate linear regression.  ?=unclear | + /−/? |
| 22 | Goodness of fit:  +=Goodness of fit was presented with either explained variance, % correctly classified, area under the curve or C-Statistic  -=Goodness of fit was not presented | *+ /−* |
| 23 | Funding Source:  += Funding source is unbiased (examples include: unfunded, government funded)  -=Funding source is biased (examples include: insurance company, or funders involved in the treatment of patients.)  ?=unclear | *+ /−/?* |
| 24 | Overall Assessment of article on a scale from 0 to 10 with 0 meaning that I do not trust the findings from this study at all to 10 meaning I trust the results from this study completely. I rate this study with | *+/-* |
|  | 0 10 |  |

References

1. Vittinghoff E, McCulloch CE. Relaxing the rules of ten events per variable in logistic and cox regression. American Journal of Epidemiology; 165(6):710-718
2. Peduzzi P, Concato J, Kemper E, Holford TR, Feinstein AR. A simulation study of the number of events per variable in logistic regression analysis. J Clin Epidemiol. 1996;49: 1373–9.
3. Hayden J, Cote P, Bombardier C. Evaluation of the quality of prognosis studies in systematic reviews. Ann Intern Med. 2006;144:427-437.

Point system: other than criteria 6 and 9 which are worth 2 points, all other criteria are worth 1 point.

Appendix III: Examples

| Question | Good Example (+) | Bad Example (-) |
| --- | --- | --- |
| Q2. Adequate description of inclusion and exclusion criteria : (Examples of important inclusion/exclusion criteria descriptions of the following (but not limited to: age, duration of symptoms, duration of sick leave, comorbidity,) |  |  |
| Q3. Recruitment setting described adequately: |  |  |
| Q4: Recruitment methods are adequately described: |  |  |
| Q5: The baseline study sample (i.e., individuals entering the study) is adequately described. Key characteristics include but are not limited or restricted to: age, gender, type of work, comorbidity, duration of sick leave) |  |  |
